# Advances Towards Synthetic Machines at the Molecular and Nanoscale Level

**DOI:** 10.3390/ijms11062453

**Published:** 2010-06-11

**Authors:** Kristina Konstas, Steven J. Langford, Melissa J. Latter

**Affiliations:** 1 School of Chemistry, Monash University, Clayton, Victoria, 3800, Australia; E-Mail: Kristina.Konstas@sci.monash.edu.au; 2 Centre for Strategic Nano-fabrication, The University of Western Australia, Crawley, West Australia 6009 Australia; E-Mail: Melissa.Latter@uwa.edu.au

**Keywords:** molecular machines, devices, switches, catenanes, rotaxanes, nanovehicles, rotary motors

## Abstract

The fabrication of increasingly smaller machines to the nanometer scale can be achieved by either a “top-down” or “bottom-up” approach. While the former is reaching its limits of resolution, the latter is showing promise for the assembly of molecular components, in a comparable approach to natural systems, to produce functioning ensembles in a controlled and predetermined manner. In this review we focus on recent progress in molecular systems that act as molecular machine prototypes such as switches, motors, vehicles and logic operators.

## 1. Introduction

A *machine* is defined by the Oxford dictionary as ‘*an apparatus for applying mechanical power, having several parts each with a defined function*’. Typically a machine requires at least one moveable component that leads to the generation of work. A molecular or nano-scaled machine can therefore be defined as an ensemble of molecular components or devices assembled to achieve a specific level of mechanical work (output) as a consequence of an external stimuli (input) [1–3]. Each molecular component performs a single function (structural or dynamic) whilst the entire assembly performs a more complex function leading to work. In a molecular system, the concept of movement (leading to work) is possible by molecular processes such as translational, rotational or geometric isomerism. Translational isomerisation involves movement of one component relative to another between two (or more) points differentiated by space. Rotational isomerism occurs in molecules (rotamers) about a specific axis if possible. This could be around a single carbon-carbon bond or in complex interlocked molecules. Geometric isomerisation particularly relates to the change in spatial orientation of two or more components constrained by an existing connectivity.

Scientists and engineers have learnt from the selectivity, specificity, precision and accuracy of biological processes and the ensembles so formed on a cellular and sub-cellular level and strive to apply these concepts in the laboratory to create molecular devices and machines. Natural machines such as ATP-ase, DNA-polymerase, opsin/rhodopsin, ribosomes *etc.* are all complex and fascinating examples of natures approach to nano-scaled machines. The synthesis and assembly of molecular building blocks capable of functioning in a controlled way and in a wholly synthetic sense is an achievable goal and, to this end, prototypical machines that demonstrate specific tasks or design features are being reported in ever increasing amounts. In fact, a simple web of science search identifies over 450 papers with the term ‘molecular machine’ in its title over the past decade.

To generalize the design principles of a molecular level machine, the following criteria have been identified as important features: 1) what energy input is required to make the machine perform work, 2) what is the type and level of movement performed by its components, 3) how will the machine’s function be detected and monitored during operation, 4) does the system have a plausible repeat operation and can this establish a recurring process, 5) what is the timescale for a complete cycle of operation, and 6) what level and type of function is to be performed by the machine [1–4].

The earliest recognition and notion of building nano-scaled artificial molecular machines and devices was made by the Nobel Laureate Richard P. Feynman in 1959 [5,6]. His presentation entitled “There is Plenty of Room at the Bottom” to the American Physical Society sparked a great enthusiasm for scientists to pursue the construction of unimaginable miniaturized complex components in a complementary way to Nature. His argument is predicated on a vision that the ‘*Large-Downward* (*Top-Down*)’ approach to the nanometer-level for such construction by dismantling elemental arrays to this level will suffer through heat dissipation, resolution and the cost of fabrication. A better approach, termed the ‘*Small-Upward* (*Bottom-Up*)’ strategy, was argued based on natural examples - starting from small molecules and building up to a nanostructure through self-assembly and self-organisation processes [7]. This approach gives chemists the upper-hand in designing and developing strategies for the construction of molecular machines and devices. This review aims to highlight some of the advances made towards functional systems capable of molecular switching, motion and transport with a long-term goal of synthetic machinery examples at the molecular level.

## 2. Interlocked Molecules – Catenanes

**Figure 1.**
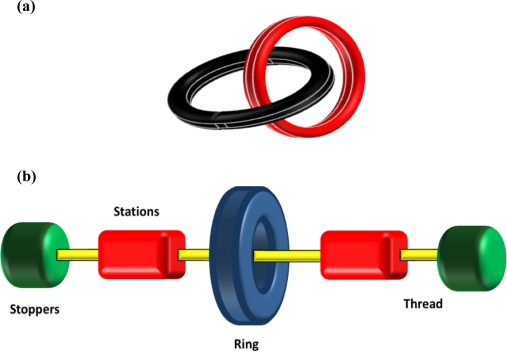
Schematic representation of two different types of interlocked molecules that have shown potential as molecular level machinery components. **(a)** Interlocked ring structures referred to as catenanes and **(b)** interlocked ring and thread terminated by bulky stoppers are termed rotaxanes.

Mechanically-interlocked molecules have attracted considerable attention as the basis of molecular devices and nanoscale machines long after the first reported syntheses of these chemical curiosities in the mid 1960’s [8,9]. Catenanes (from the Latin *catena* meaning chain) are the most common of this class and are defined by their interlocking molecular rings (Figure 1a). Rotaxanes (*rota* meaning wheel, *axis* meaning axle) are another common form (Figure 1b). With respect to nomenclature, the number (represented as n) is enclosed in square brackets, [n] and describes the number of molecular parts involved in mechanical bonding. For example, a [2]catenane denotes two interlocking rings, a [4]catenane denotes four interlocking rings.

The molecular dynamics of each component with respect to the other can be controlled and has been shown to be the basis of simple molecular devices. Recent developments in synthetic paradigms (template-direction etc) has allowed for the preparation of a variety of catenanes and more complex structures, such as trefoil knots in high yield [1,10–19]. The preparation of interlocked molecules involves two integral steps: (i) the molecular recognition and arrangement of the non-interlocked components in a threaded fashion – so-called self-assembly, and (ii) covalent bond forming reactions post molecular recognition, which mechanically-interlocks the two components. The syntheses of interlocking molecules were once deemed a great synthetic challenge, with often more than 20 multiple-step syntheses being employed using the covalent approach and ultimately leading to a statistical result with yields of less than one percent [9,20,21]. Synthetic strategies based on various non-covalent templates have been proposed by several research groups, leading to efficient preparative procedures with yields typically above 50%. Synthetic template-directed strategies are based on non-covalent interactions, such as hydrogen bonding, donor-acceptor interactions and metal complexation, which are used to assemble a pre-organized macrocyclic component and a linear component in a threading process, which after a bond-forming reaction, affords the interlocked molecules [22,23]. In addition, knotted interlocked molecules have also been prepared from single-stranded DNA amongst other molecules [24–26]. Of the non-covalent templates described in the literature, the most promising synthetic strategy are those utilizing transition metal templates or complementary π-electron-rich and π-electron-deficient recognition sites.

**Figure 2.**
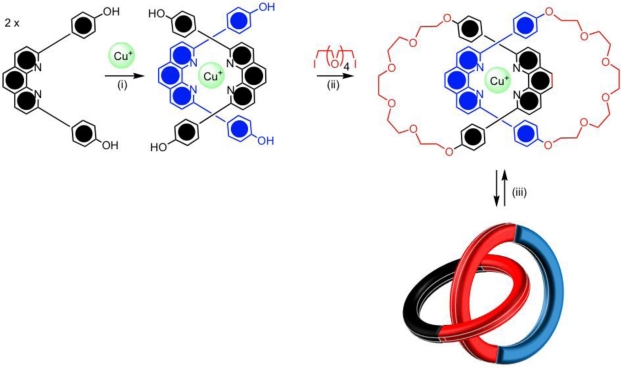
Sauvage’s copper(I) template synthesis of an interlocked catenane in two sequential steps: **(i)** coordination of the disubstituted phenanthroline ligand to the metal; **(ii)** mechanical interlocking by a covalent bond forming reaction; **(iii)** Demetallation leads to a specific conformational change.

The metal ion template strategy was first developed by Sauvage and co-workers using copper(I) ions and phenanthroline ligands [27,28]. The shape and mutually orthogonal arrangement of the disubstituted phenanthroline ligands would encapsulate the tetrahedral copper(I) metal ion, generating the necessary cross-over point for catenane formation giving a *pseudo*-tetrahedral complex (Figure 2(i)). With the introduction of hydroxyl groups on the phenyl substituents of the phenanthroline ligand, the half-rings could undergo a Williamson ether macrocyclization reaction, as seen in Figure 2(ii). This synthesis provided a simplistic and efficient means for preparing catenanes but also rotaxanes and to a smaller extent knotted molecules [10]. By changing the oxidation state of the metal, or by competing ligation, the metal ion could be removed to give the free catenane. Sauvage’s phenanthroline catenane undergoes a co-conformational change, which involves the circumrotation of both the macrocycles through the cavity of each other upon demetallation, leading to a conformation in which the phenanthroline ligands to be positioned away from each other (Figure 2(iii)). This is an example of a chemically controllable catenane.

**Figure 3.**
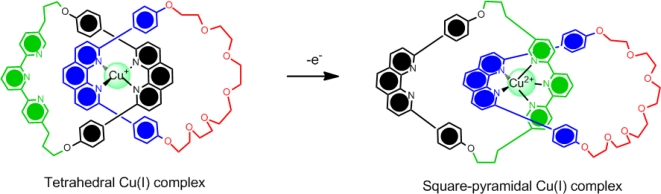
A model for a catenane-based molecular switch that is redox-switchable by control of the copper oxidation state [29].

In addition, the co-conformational motion can also be controlled electrochemically by reversible oxidation/reduction reactions of the metal centre and judicial choice of the ligands. For example, while copper(I) prefers a four-coordinate environment, copper (II) prefers a five-coordinate environment leading to ligands capable of stabilizing this ion upon oxidation. The example shown in Figure 3 is a model for a molecular switch based on a [2]catenane [29]. This model demonstrated in earlier work by Sauvage uses one of the macrocyclic rings to contain two discrete binding sites on opposing sides of the ring–terpyridyl (terpy) and diphenylphenanthroline (dpp). The second macrocyclic ring contains only a single dpp binding unit. Upon changing the oxidation state of the copper(I) to copper(II), the dpp/terpy conformation is adopted to favor the five-coordinate copper(II) preference. These manipulations have lead to the formation of molecular switches and muscles [30–33].

**Figure 4.**
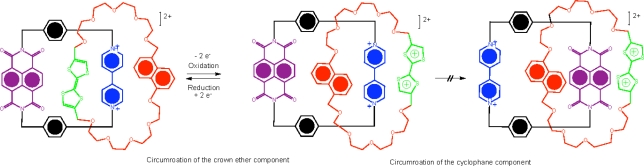
A representation of the desymmetrized donor-acceptor [2]catenane [34].

Sauvage’s catenane is an early example of the processes that can be applied to interlocking molecules but a more amenable approach to switching behavior leading to rotational isomerism involves desymmetrization of the [2]catenane within the different ring components [34]. The synthesis of such compounds is challenging as they require the selection of pairs of complementary recognition units capable of distinctive binding strengths in both the neutral and reduced/oxidized states. The use of π-donors, namely tetrathiafulvalene (TTF), 1,5-dioxynaphthalene (DNP) and naphthalene diimide (NDI) moieties have been incorporated in a crown ether macrocyclic component that selectively holds the π-deficient tetracationic cyclophane around the donor TTF station. Rotation of the crown ether components in the TTF-containing [2]catenane-NDI interlocked molecule can be driven chemically, by oxidation and reduction cycles leading to a switching mechanism with an optical readout [34]. In this case, no translocation of the dicationic cyclophane occurs in response to the oxidative switching of the doubly charged species and the NDI unit remains outside of the electron rich crown ether as verified by ^1^H NMR spectroscopy (Figure 4). These types of [2]catenane molecules are appealing and have the potential to act as machine-like molecules based on the movement of one component relative to the other, or as a molecular electronic device based on the electronic switching, a topic described in greater detail in Section 4.

**Figure 5.**
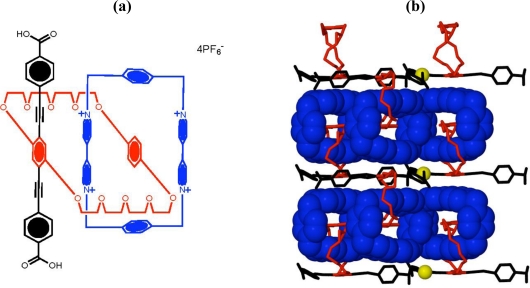
**(a)** Stoddart and Yaghi’s catenane used in a metal-organic framework, **(b)** Side on view of a portion of the MOF along the *a*-axis [37].

There are two significant challenges that need to be overcome in order to achieve a pragmatic molecular device or machine. While the catenanes discussed already display machine-like processes, it is all achieved in solution with properties of a bulk nature being measured. As with natural systems, most machines are housed upon a platform or scaffold of some sort, be it a membrane, cell wall, or macroscopically, a factory floor. Furthermore, machines tend to be well-ordered so that their action and output can be used for work either as a sole entity, or in concert with other machine processes. There has been much achieved to date in each individual area, however few examples attempt to solve both necessities. One of the more recent attempts to overcome these challenges, involves embedding the catenanes into a metal-organic frameworks (MOF) in which the crystal lattice is utilized as the solid support [35,36]. The amalgamation of Stoddart and Yaghi’s research has now led to new developments in metal-organic frameworks with a donor-acceptor catenane (Figure 5(a)) being crystallized with copper(I) to give a solid-state 2D network (Figure 5(b)) [37]. This structure demonstrates the first step towards the construction of new synthetic materials that contain dynamic behavior into robust crystalline scaffolds.

## 3. Interlocked Molecules – Rotaxanes

Rotaxanes differ from catenanes in that they are composed of both ring (cyclic) and axle (acyclic) components. A [2]rotaxane is formed when a thread, dumbbell or axle component is mechanically interlocked with a ring component. If the axle component is stoppered at both ends with bulky functional groups to prevent disassociation of the ring component at ambient temperature (Figure 1b), the system is called a rotaxane. If there are no stoppers or the stoppers are not of sufficient size to preclude dissociation at ambient temperature, the molecular assembly is called a [2]pseudorotaxane. A [3]rotaxane is typically formed when two rings are threaded onto an axle component, though two axles and one ring also satisfy the definition. There are three common methods to prepare rotaxanes, namely *capping*, *clipping* and *slipping* but a more recent improvement *via* a template effect has improved yields over what was previously achieved by statistical synthesis. The worth of rotaxanes as a molecular form of abacus or transport agent and their capacity to shuttle (molecular motion) and switch (molecular logic) has seen research in the area flourish over the past decade and much effort has been dedicated to advancing synthetic methodologies for enhanced functions [1,38–42].

**Figure 6.**
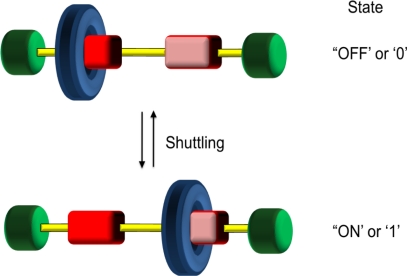
Rotaxanes are able to ‘shuttle’ between two different states indicating switching (ON/OFF) or binary processes (0/1). Shuttling is a form of translational isomerism. The challenge is controlling this motion.

The use of rotaxanes as a prototype for a class of molecular machines has arisen as a result of their ability to undergo translational isomerisation between two or more structures (Figure 6). This isomerisation is a result of the translational molecular motion of the macrocyclic ring along the acyclic component. The equilibration and dynamic behavior back and forth along the acyclic component is also known as *shuttling* in the same way a bus or tram may move between two stations at an airport. This shuttling can take place up to 40,000 times per second. Molecular recognition sites are built into both components to produce thermodynamic sinks resulting in points along the linear component where the two or more components predominantly reside through a bias. Again, an electronic complementarity between the components is vital for both synthesis and function. This complementarity often means that rotaxanes are often highly colored species.

**Figure 7.**
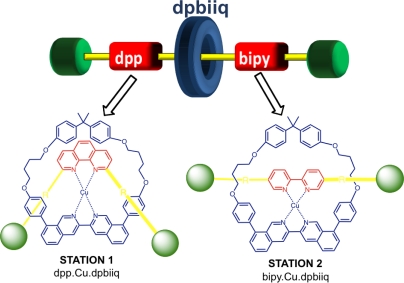
Copper-complexing [2]rotaxane comprising two different bidentate stations [43]. The diphenylbiisoquinoline ligand (dpbiiq) site in the macrocyclic ring structure is complementary to either the diphenylphenanthroline (dpp) or bipyridine (bipy) sites along the threading molecule and shuttling is possible upon electrochemically-driven complexation events with copper.

Metal complexing sites, quite often of different denticity (*i.e.*, bidentate and tridentate) have been a popular choice for “stations” on axle components, with molecular shuttling driven by electrochemical stimulation. Sauvage and co-workers have since shown stations of the same denticity may also be incorporated into the axle structure and function as a [2]rotaxane *via* electrochemical means [43]. In this example, the axle binding sites include a highly shielding phenanthroline ligand and a non sterically hindering bipyridine chelate while the complementarity to afford copper complexation is provided by a bisquinoline unit in the macrocylic ring (Figure 7). Although the preparation involves a multi-step synthesis, the electrochemical-induced shuttling between the two stations is a fast, clean process and shows promise for multi-state machine developments in the future.

**Figure 8.**
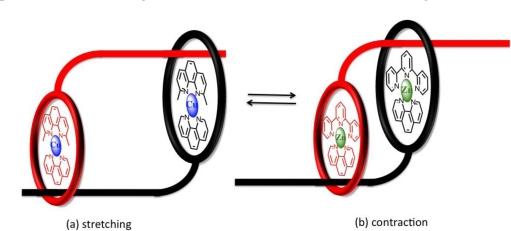
Schematic representation of Sauvage’s “artificial muscle” using transition metal complexation to interchange between **(a)** stretched and **(b)** contracted geometries.

Extending the topology and complexity of linear motors, the stretching and contraction of skeletal muscle may also be replicated in a synthetic sense by one-dimensional molecular assemblies acting as *molecular muscles* upon the action of an external stimuli [44–48]. Using a transition metal complexation strategy involving a symmetrical, double-threaded topology Sauvage was able to demonstrate “artificial muscle” processes with changes in backbone length approximated to be between 65–85Å from molecular modelling. The key to their system was the use of two [2]rotaxanes to form a dimer, with this orientation allowing the individual thread of each rotaxane to move along the other, interconverting between a contracted and stretched confirmation akin to the working action of skeletal muscles. Each thread was synthesized to contain both bidentate and tridentate chelate “stations” with exchange between copper(I) and zinc(II) respectively facilitating motion (Figure 8) [49].

A more recent example of a molecular muscle reported from the Chiu research group utilizes a molecular cage that acts as the ring component of the [2]rotaxane with the additional muscle function possible by threading an axle component that is able to contract and stretch upon fluoride addition or removal respectively [50]. In their system, an axle bearing two ammonium stations, one pyridinium station and terminated by an alkyl bromide is threaded through the macrocyclic cage. Reaction of the threaded axle with a substituted pyridine yields a [2]rotaxane where the original thread reacted with the substituted pyridine to incorporate a fourth station (a second pyridinium). The new [2]rotaxane, comprised of the macrocyclic cage and thread with four stations (two ammonium stations and two pyridinium stations) was observed by NMR spectroscopy to stretch and contract through anion exchange. One of the biggest challenges in trying to mimic any natural processes in the laboratory is replicating the efficiency of the process so possibly of greater significance is the reported change of 36% between contracted and stretched conformations (based on molecular dynamic simulations), which is greater than the 27% change in human muscle.

Extending from the concept of linear motion demonstrated by rotaxane based machines that shuttle between multiple stations, Stoddart and co-workers have progressed this research area further to pioneer a two component machine that mimics the behavior of an elevator at the molecular level [51]. One component of the machine is comprised of a central aromatic platform appended with feet forming a tripod type arrangement. Incorporated within each of the three feet are two different binding sites: at the upper level, ammonium binding sites, and bipyridinium at the lower level both of which are able to interact with each of the three complementary crown ethers of the second component of the elevator, utilizing the knowledge and success of this binding motif in many of Stoddart’s classical rotaxane examples [10]. Stoddart reports the molecular elevator has an approximate diameter of 3.5 nm and height of 2.5 nm with vertical movement (estimated distance of 0.7 nm) possible through acid-base manipulation [51]

**Figure 9.**
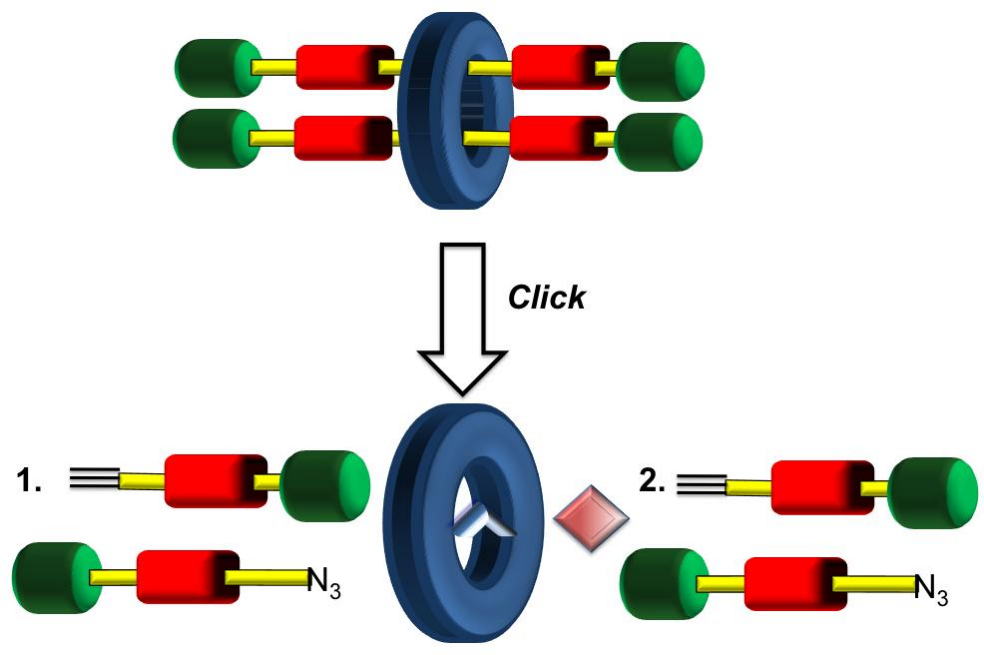
Template-assisted, one pot synthesis of a [3]rotaxane. Two, successive covalent bonds are formed (*via* the CuAAC click reaction) assisted by ligation to the ring yielding an interlocked structure consisting of two threaded components [53].

What should be obvious to the reader by now is the essential requirement of multiple stations positioned along an axle for shuttling to occur in rotaxane machinery. As this research area has flourished with examples of such linear motion, some pivotal milestones have seen marked improvements with respect to molecular complexity (increased topology and threading). An even more recent development from the Leigh group has seen complexity rise further whereby more than one axle component is threaded through the same macrocycle yielding a [3]rotaxane in one pot as shown schematically in Figure 9 [53]. The success in achieving this more complicated interlocked system arises from the design considerations of the macrocycle. The positioning of a ligand in the marcocyclic structure functions in two ways: (1) act as a ligation site for a transition metal that will act as a catalyst in the bond formation of the axle components, and (2) provides a template for successive covalent bond formation of interlocked axle components. This high yielding methodology may be applicable to other higher order interlocked molecular assemblies which is important as molecular machinery design continues to develop in terms of complexity.

**Figure 10.**
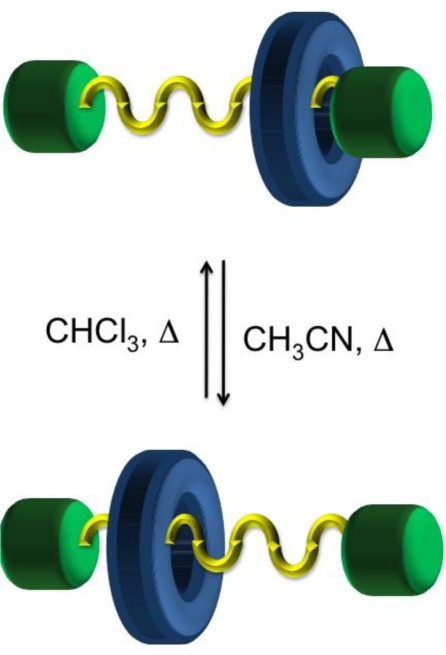
Schematic representation of a [2]rotaxane molecular machine that is comprised of an oligopeptide axle that results in a helical structure.

Interest has also extended to the potential biological applications mechanically interlocked rotaxanes may offer through the encapsulation or protection provided by the macrocyclic ring about the axle component. In particular, Leigh and co-workers reported a rotaxane comprised of a pentapeptide axle and macrocycle that protects against peptidase-catalysed hydrolysis over several days [54]. The use of oligopeptides axle components was also reported by Moretto *et al.* and the characteristic shuttling between stations was shown upon changing solvents (Figure 10) [55].

## 4. Switches and Molecular Logic Gates

One variety of a molecular switch can be described as a nanoscale machine which undergoes reversible co-conformational shifts or molecular transformations (leading to an output) in response to protons, photons, electrons, temperature, or exogenous cations or anions (as inputs) between two or more states [56]. We have already seen simple prototypes of this design in the previous sections. As molecular switches convert input stimulations into output signals, these systems can be seen as performing simple logic operations, that is through ON/OFF or ‘0’ and ‘1’ type processes [57]. The use of two or more switches or gates can lead to Boolean operations including addition and subtraction. While simple molecular systems have led to displays of the simplest functions, such as YES or NOT, ‘1’ is only with the advent of molecular recognition and self assembly, that mechanically interlocked molecules and supramolecular arrays have been efficiently used to prepare more complex logic gates including AND, XOR and INHIBIT. This section provides a small selection of supramolecular switches and logic gates.

**Figure 11.**
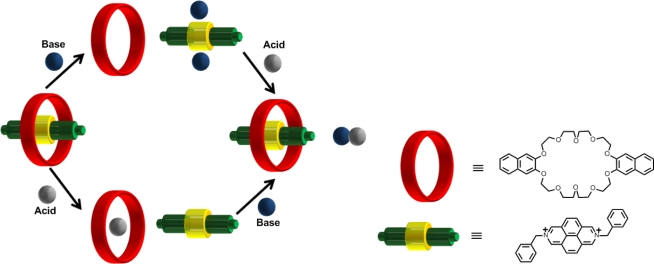
A schematic diagram of the unthreading and threading of a [2]pseudorotaxane, corresponding to an XOR logic function [58].

One of the earliest examples is that of a molecular XOR logic gate defined by the inclusion and exclusion of the two components of a [2]pseudorotaxane [58]. In this early example, Figure 11, the [2]pseudorotaxane contains a good electron acceptor (2,7-diazapyenium) which interacts with the electron donating group from an aromatic crown ether. The pseudorotaxane self-assembles due to the π-electron-acceptor-donor interactions of the two groups. In this case the unthreading and treading of the [2]pseudorotaxane occurs with the use of an acid and base stimuli. Addition of a suitable base (tributylamine, DABCO) unthreads the pseudorotaxane and forms the acceptor(base)_2_ complex and with the addition of acid the pseudorotaxane is formed again along with a salt.

**Figure 12.**
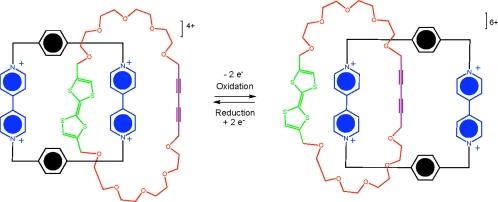
A representation of the push-button molecular switch [59].

As a result of advancements made towards the synthesis of interlocking molecules, several rotaxanes and catenane–based molecular switches have been developed. Several interesting assemblies have been recently published, one of which demonstrates a [2]catenane molecule which operates as a single push-button molecular switch [59]. The resulting mechanically interlocked molecule exists in one translational form in its ground state that is mechanically switched to another translation form in the excited oxidized state (Figure 12).

**Figure 13.**
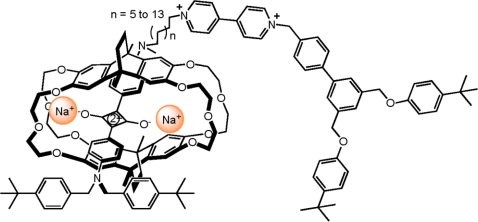
A representation of the squaraine-based [2]rotaxane molecular switch of *Chiu* [60].

Recently developed by *Chiu* is this visible-active squaraine-based [2]rotaxane molecular switch (Figure 13) [60]. This molecular switch operates through the selective encapsulation and exposure of the squaraine station of a two-station [2]rotaxane upon the addition and removal of sodium ions, respectively. Long-waved-fluorescence signals are produced and can be seen when the sample is irradiated with 365 nm UV light. The co-conformational state is reversibly upon switching with the addition of a sodium ion source and in this particular example sodium perchlorate was used for this purpose.

## 5. Molecular Rotary Motors

**Figure 14.**
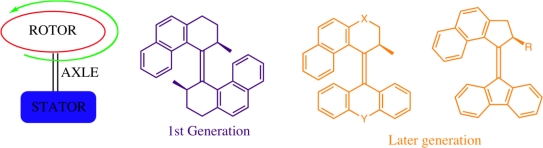
Schematic representation of the general design components of the Feringa rotary molecular motor with evolutionary design [61].

Biological motors consume chemical energy that drive sequential changes with other molecules to perform highly specialized tasks and functions essential for life. The design principles that facilitate the complex and precise actions of such motors is a significant challenge for their successful application in synthetic environments [61,62]. The following two sections will describe efforts to create rotary motors followed by translation motion towards the transport of cargo at the molecular level. The Feringa laboratory have been successful at demonstrating repetitive, unidirectional rotation (of MHz frequencies) using organic frameworks which have a fixed stator component connected to a rotor through an axle which operate upon external stimulus [63–65]. Exploitation of the light induced *cis*-*trans* isomerisation has proven to be a popular design and a variety of rotary motors are possible through combinations of different organic frameworks flanking a central alkene axle (Figure 14). The upper and lower organic units may be identical (1^st^ generation rotary molecular motor) or differ in structure (2^nd^ generation rotary molecular motor) and choice of substituents may further assist in controlling the rotational speed of these motors through steric interactions [66,67].

**Figure 15.**
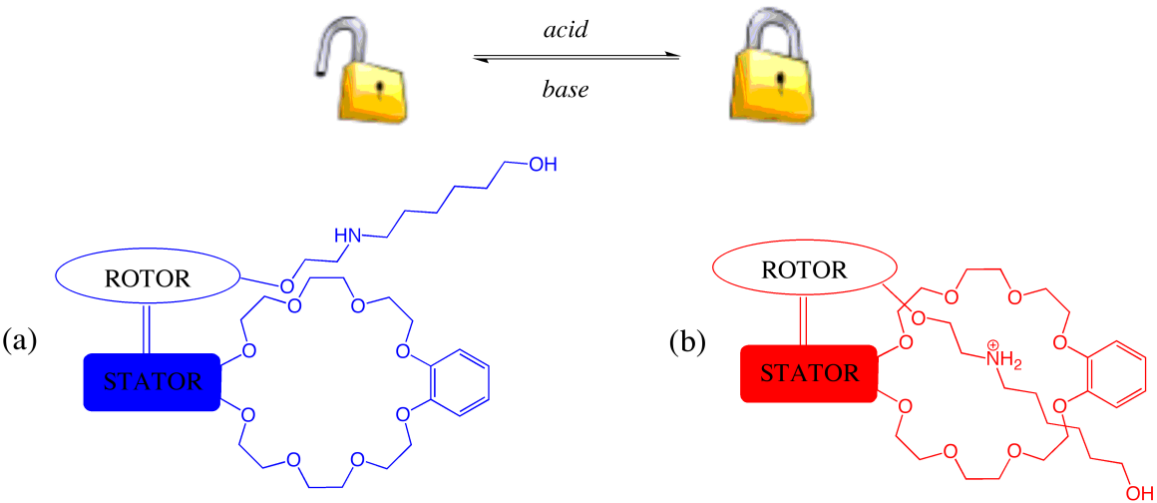
Schematic representation of a molecular rotary motor with self-complexing lock. The well known complementarity between crown ethers and ammonium salts provides a non-covalent interaction to produce a (a) locked and (b) unlocked state for the molecular rotary motor.

A recent development in controlling the rotary motion of the Feringa motors involved the introduction of a self-complexing lock which functions in a similar way to the rotaxane threading process (see Section 3) [68]. As shown schematically in Figure 15, the rotor component has been covalently modified with a functionalized thread while the stator was appended with a macrocyclic crown ether ring. The threading process is under acid-base control due to the non-covalent interaction between the crown ether and ammonium salt in its protonated state and results in a *locked* or *unlocked* state for the rotary motion of the rotor component.

## 6. Molecular Transport

**Figure 16.**
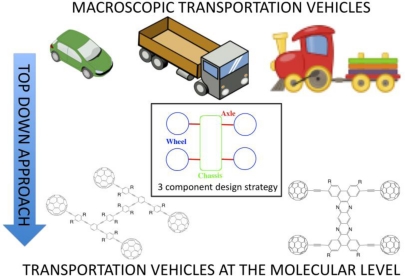
The concept of a “top-down” approach for the construction of vehicles at the molecular level as developed by the Tour group. Using a three component design strategy analogues of macroscopic transportation vehicles (e.g., cars, trucks, trains) have been created such as nanocars and nanotrucks. R = solubilizing groups.

The ability to control mechanical motion and potentially transport cargo are challenges being actively explored [69–71]. The original report on single-molecule nanocars detailed the synthesis of a semi-rigid organic chassis appended with four fullerene wheels *via* alkynyl axles which moved in a directional rolling motion across a gold surface (observed by scanning probe microscopy) [72]. While the nanocar was a significant achievement in establishing proof-of-concept for the ability to roll (rather than slide) molecular wheels across a surface (upon heating the gold surface) it also highlighted the essential design components to include: (1) a chassis, (2) multiple wheels and (3) axles which link the wheels to the chassis (Figure 16). Subsequent modifications and improvements with regards to these three components has since paved the way for later generation nanovehicles [71].

Modification of the chassis fragment to incorporate a planar polyaromatic spacer yielded a construct referred to as a nanotruck which was designed to provide a suitable “loading bay” for either acid or base binding to the aza-aromatic spacer although STM imaging to demonstrate transport across a metal surface was hindered by surface impurities and possible stability issues relating to surface chemistry [69]. Another structural variation of the nanotruck has been prepared and is comprised of a porphyrin core with carborane wheels [73]. If future surface imaging confirms the expected rolling motion, this nanotruck also has the ability to transport metals *via* coordination within the porphyrin macrocycle and may realize the goal of cargo delivery between specific locations. Improvements have also included the replacement of fullerene wheels to include ruthenium-based wheels [74] which not only improve solubility compared to the fullerenes but are also less restrictive in synthetic manipulations.

The Feringa unidirectional motor (see Section 5) as powered through light stimulus was chosen to power the ‘nanocar’ but due to incompatibilities with the original fullerene wheel design was adapted to an improved nanocar model with carborane wheels [75]. Three-wheeled nanocars [69] and pinwheel [76] arrangements have also been synthesized and characterized fully. While such designs offer limited translational motion they show much greater potential for a pivoting motion about a central axis, although conclusive imaging data to support this has not been reported to date. To truly ‘make the molecules do the work’ at the nanoscale and produce nanovehicles in an assembly line type manner self-assembled structures have also been examined in this research program [77].

The biggest advantage to such approach lies in the reduced synthetic effort, which for the examples discussed above is multi-step, time consuming, low yielding and even with the modular, three component design protocol does not allow for structural modification as easily as a self-assembled approach. Both metal coordination and hydrogen bonding interactions were trialed to assemble fragments successfully into carborane-wheeled ‘nanocars’ although stability under surface imaging conditions has yet to be determined. The assembly line methodology has also been extended to create a linear nanotrain through the assembly of multiple fragments of a 2-pyridone which contains two hydrogen bonding site located on opposite ends of the monomer [78]. It seems a logical development that the assembly line could also be a useful approach for assembly ‘carriages’ of varying components which individually could serve different functions to the transportable vehicle.

**Figure 17.**
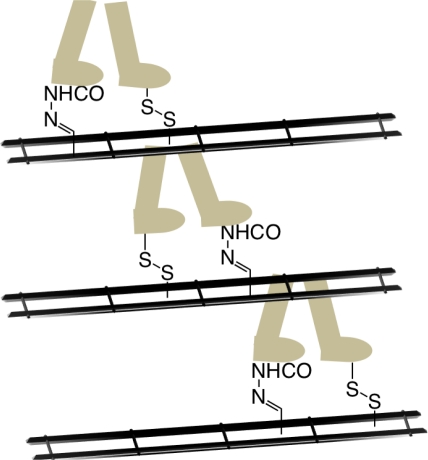
Teaching molecules to walk. The use of a scaffold containing functionality designed to make disulfide and hydrazone links has been used to allow a molecule to transverse the walkway in a controlled fashion.

An advancement on molecular motion in a different structural form to Tours nanovehicles arose from the Leigh laboratory recently where a small molecule (synthetic) was shown to walk along a track comprised of four foot holes in a repetitive manner through changes to the chemical environment (Figure 17) [79]. The walking motion is possible due to careful design of the interactions between the feet and track. Specifically, the use of disulfide and hydrazone units allows for kinetic control between the feet and track upon different chemical stimulus (acid or base). Taking inspiration from biological motor proteins further studies are being conducted to investigate whether the walking molecule is able to carry and deliver a cargo and over what distance this linear motion may be achieved by extending the track using polymeric units.

## 7. Conclusions

In reviewing the current literature relating to molecular machine research we aimed to illustrate fundamental developments and progress relating to typical functions carried out by macroscopic machinery including transport, rotation and switching. While each of those alone does not create an artificial machine the progress does make a significant contribution to unlocking the key processes relating to control and function at the molecular level. Biological molecular machines far outweigh any artificially constructed machine both in complexity and function which will continue to inspire and challenge scientist for many years to come, one that will no doubt benefit through multi-disciplinary cooperation.
